# Ultrasound-guided antegrade brachial artery access closure: a staged withdrawal technique for improved hemostasis

**DOI:** 10.1186/s42155-025-00591-6

**Published:** 2025-12-02

**Authors:** Sabharisundaravel Paulraj, Anadi Gupta, Rohit Khandelwal, Sayantan Patra, Sreeni Sivan Pillai, Shuvro H. Roy-Choudhury

**Affiliations:** 1 Department of Interventional & Endovascular Radiology, R.N. Tagore Hospital, Narayana Health, Mukundapur, Kolkata, West Bengal India; 2 University Hospitals of North Midlands NHS Trust, Stoke-On-Trent, UK

## Abstract

**Supplementary Information:**

The online version contains supplementary material available at 10.1186/s42155-025-00591-6.

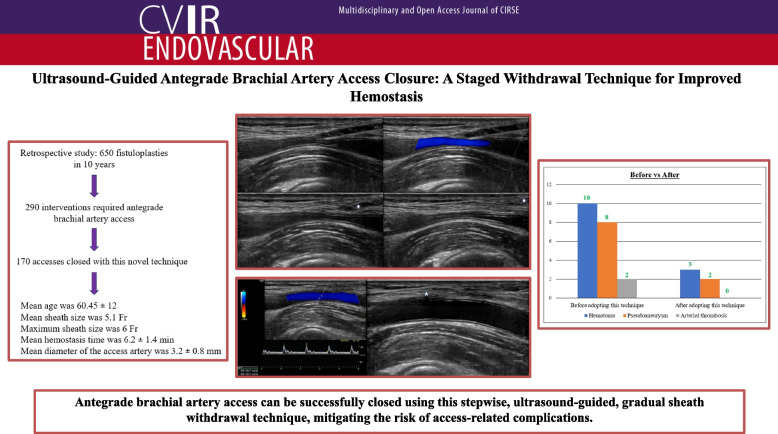

## Introduction

Brachial artery access is increasingly utilized for endovascular procedures, particularly when femoral or radial access is deemed less suitable or unavailable [1–4]. Owing to its superficial course and favorable caliber, the brachial artery serves as an appealing route for interventions involving upper limb vasculature, dialysis access circuits, and complex aortic procedures.

At our center, antegrade brachial access is increasingly favored for challenging forearm arteriovenous fistula (AVF) interventions, particularly those involving the perianastomotic lesions, as has been reported in other single-center experiences [5]. A perianastomotic lesion in a distal forearm radiocephalic fistula is typically treated with radial artery or venous access. This is predominantly because brachial artery access is associated with a high degree of local complications [6], purportedly due to the lack of a suitable flat or hard surface (e.g., femoral head in common femoral arterial access) to compress against. However, antegrade brachial artery access offers several advantages, including cannulation aligned with the direction of arterial blood flow, the ability to treat the entire circuit from a single access, plaque redistribution in line with flow after angioplasty, ensuring a smooth transition from the arterial to the venous segment and promoting more physiological, laminar flow dynamics, compared to radial artery access [7].

To enhance the safety of antegrade brachial artery access during arteriovenous fistula interventions, we implemented a simple ultrasound-guided, staged sheath withdrawal technique designed to create a plug at the arteriotomy site and minimize access-related complications.

## Methods

This single-centre retrospective analytical study was conducted between May 2014 and February 2024. Due to its retrospective nature, formal Institutional Review Board approval was waived; however, written informed consent was obtained from all patients prior to the procedure. Data were collected through a retrospective analysis of a prospectively maintained database and a review of electronic medical records. Among 680 fistuloplasties performed, 290 required antegrade brachial artery access, only for distal forearm radiocephalic fistulas with perianastomotic lesions. The ultrasound-guided staged sheath withdrawal technique was developed and adopted in the latter half of this period, with 170 consecutive patients undergoing access site closure using this method. Complication rates before and after adoption of this technique, were compared using Fisher’s exact test. A *p*-value < 0.05 was considered statistically significant.

**Fig. 1:**
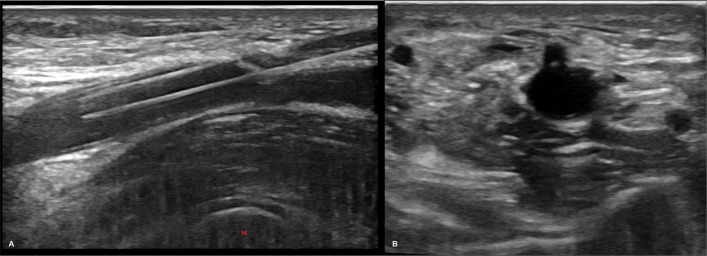
Baseline ultrasound assessment following arteriovenous fistula intervention. **a** Longitudinal grayscale ultrasound image showing a 5 Fr sheath positioned within the brachial artery, aligned directly over the medial epicondyle (denoted by ‘M’). **b** Transverse ultrasound image confirming central placement of the sheath at the 12 o’clock position relative to the arterial lumen

Patients were positioned supine with the access arm abducted at 90 degrees. Preprocedural ultrasound was used to evaluate brachial artery diameter and detect calcifications. The puncture site was chosen proximal to the brachial bifurcation and aligned over the medial epicondyle, enabling guidewire advancement into the radial artery as well as giving some bony protuberance to press against. In patients with a high brachial bifurcation, arterial access was instead obtained from the radial artery at the level of the medial epicondyle. Arterial entry was consistently performed at a 45-degree angle targeting the 12 o’clock position, as confirmed on the transverse ultrasound plane (Fig. 1).

Arterial access was achieved using a 5 Fr micropuncture introducer set (Cook Medical Inc, Bloomington, Indiana, USA) with a 21G needle and a 0.018-inch guidewire, following local anaesthetic infiltration under real-time ultrasound guidance. A 5 Fr sheath (Cordis AVANTI® +, Miami Lakes, Florida, USA) was used in 275 cases; however, sheath size was upsized to 6 Fr sheath (Cordis AVANTI® +, Miami Lakes, Florida, USA) in 15 cases to accommodate larger balloon angioplasty. Immediately after sheath placement, all patients received 3000 IU of unfractionated heparin and 100 µg of intra-arterial nitroglycerin. No additional intra-procedural antiplatelet agents or vasodilators were administered. Heparin was supplemented with 1000 IU every hour thereafter, as appropriate.

### Hemostasis technique

**Fig. 2:**
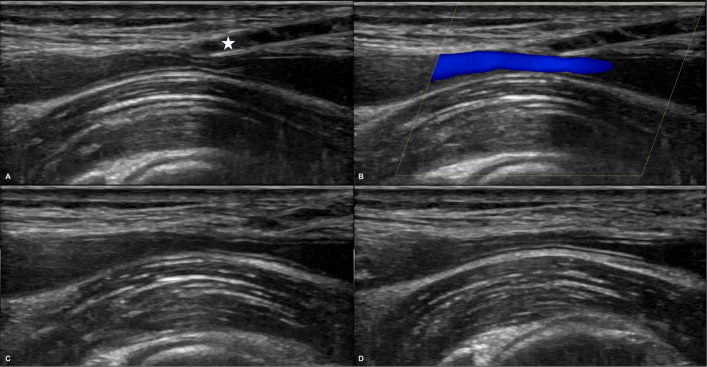
Stepwise demonstration of the staged sheath withdrawal technique under ultrasound guidance. **a** Grayscale image showing the sheath (white star) partially withdrawn to just outside the arterial adventitia, with gentle probe compression creating a subtle indentation on the brachial artery wall. **b** Compression is modulated to preserve a narrow antegrade flow column across the arterial lumen. **c**, **d** Sequential withdrawal of the sheath in 5 mm increments is performed every 30 s, following identification of a developing mural thrombus (not shown), to promote progressive tract closure

Following completion of the AVF intervention, sheath removal was initiated using a standardized ultrasound-guided staged withdrawal protocol designed to minimize access complications. Under real-time ultrasound guidance, the vascular sheath was initially withdrawn to just outside the adventitia of the access artery (Supplementary video). The partially withdrawn vascular sheath then acts as a temporary mechanical barrier, limiting blood extravasation at the puncture site. Controlled under vision, probe-mediated compression was then applied for 5 min to approximate the arterial walls, just enough to preserve a narrow residual flow column, critical for maintaining perfusion and patency of the downstream arteriovenous fistula (Fig. 2).

After the initial 5 min of ultrasound-guided compression, a focal mural thrombus was typically observed in the wall of the artery, but not protruding into the lumen at the puncture site, contributing to hemostasis. Compression was then maintained for an additional minute to stabilize the thrombus and promote plug formation. Subsequently, the sheath was withdrawn in 5 mm increments at 30 s intervals, enabling stepwise tract closure under controlled conditions. This stepwise technique also encouraged progressive clot formation along the access tract. At the end of the procedure, a thin, thread-like clot was sometimes noted at the skin puncture site. Special care was taken to avoid dislodging this clot during subsequent cleaning and dressing, as inadvertent disruption could result in re-opening of the arteriotomy site. This staged withdrawal technique was performed by four interventional radiologists with 5 to 22 years of IR experience or by trainees under direct supervision. All were trained in the protocol before implementation.

**Fig. 3:**
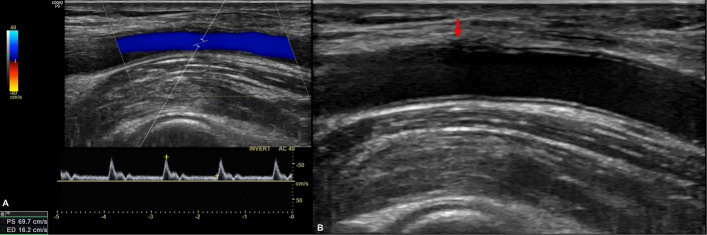
Ultrasound assessment following completion of haemostasis. **a** Spectral Doppler evaluation at the access site showing biphasic, laminar flow with a peak systolic velocity of 70 cm/sec. **b** Grayscale ultrasound image demonstrating a closed arteriotomy site (red arrow) with preserved arterial lumen and no visible hematoma or pseudoaneurysm

Technical success was defined as complete hemostasis without hematoma or pseudoaneurysm formation, preservation of distal perfusion confirmed by Doppler ultrasonography, and no requirement for re-intervention or surgical repair (Fig. 3). Following the procedure, a light compressive dressing was applied using sterile gauze and an elastic bandage (3 to 4 wraps). The access limb was immobilized using a padded arm board or a broad arm sling for a minimum of 4 h to support hemostasis. All patients underwent ultrasound evaluation of the access site within 4 to 6 h post-procedure and again at 24 h prior to discharge. Additional imaging was performed in cases of clinical concern for complications. The supplementary Video offers a detailed, step-by-step demonstration of the technique, clearly illustrating its procedural simplicity, reproducibility, and clinical effectiveness.

Before this technique, a conventional manual compression of the brachial artery access was performed after removing the access sheath at the end of every procedure by the physician completing the procedure, or, trained IR nurses under physician supervision without ultrasound guidance for 10 to 15 min until hemostasis, in the post procedure observation room. Ultrasound was used as and when necessary, particularly, if there were concerns for a hematoma, ongoing bleeding and occasionally used to complete the compression. The same protocol of post procedure surveillance using ultrasound was followed in all cases.

## Results

Baseline characteristics of both cohorts are displayed in the Supplementary Table. Technical success of this technique was 97.1% and the mean hemostasis time was 6.2 ± 1.4 min. No major access related complication requiring surgical/endovascular intervention or prolonged hospitalization intervention was seen, post adoption of this technique. Table 1 provides a comparative summary of the observed complications. Post adoption of this technique, there was significant statistical reduction in complications including hematoma (8.3% vs 1.8%, *p* = 0.010) and pseudoaneurysm formation (6.7% vs 1.2%, *p* = 0.018). No access site infections, nerve injuries, or access-related mortalities occurred during the study.

**Table 1:** Access site complications and management before and after adoption of the ultrasound-guided staged sheath withdrawal technique

Complication	Before adoption of the technique (*n* = 120)		After adoption of the technique (*n* = 170)		*p*-value
	Incidence	Management	Incidence	Management	
Hematoma	10	Conservative	3	Conservative	0.010*
Pseudoaneurysm	8	Conservative: 6 Thrombin injection: 2	2	Conservative	0.018*
Arterial thrombosis	2	Surgical embolectomy	0	–-	0.170*

^*^Significant values are indicated by asterisks (*p* < 0.05)

Conservative management of hematoma involved ice pack application, a light compressive dressing, and limb immobilization for 4 to 6 h. Pseudoaneurysms managed conservatively were initially treated with ultrasound-guided compression for 10 to 15 min until hemostasis was achieved, followed by compressive dressing and limb immobilization for 4 to 6 h. Prior to adoption of this technique, 2 out of 8 pseudoaneurysms required thrombin injection and 2 cases of arterial thrombosis required surgical embolectomy.

All patients with perianastomotic thrombotic occlusion were started on antiplatelets and/or anticoagulation prior to intervention, unless contraindicated while patients with stenotic lesions were started on single or dual anti platelets depending on age. The exact documentation of the groups is not available for this manuscript, but, given large baseline numbers, they are likely to have been equally distributed between the two groups. No difference in hemostasis times or outcomes were seen in patients on anticoagulation versus those who weren’t. We didn’t encounter any patients with abnormal coagulation status.

## Discussion

Despite its advantages, brachial artery access carries the highest complication rates among arterial access sites. Overall complication rates as high as 36% have been described for brachial arterial access with major complications as high as 7% to 11% [6]. Conventionally, therefore, brachial artery access is avoided in radiocephalic arteriovenous fistula interventions in favor of retrograde venous access into the outflow vein to treat across the anastomosis. Using a technique that might improve the safety of brachial artery access may therefore make it advantageous. Also, the use of ultrasound guidance to choose a suitable site of access (similar to us) in the brachial artery has been reported previously to reduce complications [6].

In cases of perianastomotic AVF occlusions, retrograde venous access is often challenging due to the absence of a catheterizable stump or the lack of flow-directed guidance. Antegrade brachial artery access, in contrast, provides a direct and effective route for intervention. It also enables intermittent angiographic assessment during the procedure, which is not feasible with venous access without additional exchanges. The brachial artery approach may facilitate safer and more efficient single-access interventions and could shift practice paradigms in the management of complex AVF.

In our institutional experience, ultrasound-guided brachial artery closure has proven to be consistently effective for both antegrade and retrograde accesses. This technique employs a combination of ultrasound guided precise point of access, mechanical tamponade by the indented sheath at the adventitia and precise ultrasound-guided compression, reducing arterial flow just enough to promote clot formation while maintaining antegrade perfusion. Importantly, total luminal occlusion is avoided, facilitating physiological platelet aggregation at the puncture site and preserving distal flow, especially critical in patients with functioning arteriovenous fistulas. The access at 12 o'clock is also crucial as this allows full longitudinal sheath visualization and luminal apposition without slipping. The technique’s controlled-flow approach likely contributes to the favorable hemostasis and safety outcomes observed. In our center, this technique has also been successfully employed with larger sheath sizes (up to 8 Fr) in select aortic interventions, albeit outside the dialysis access context. We have also tested this technique in common femoral punctures; however, conventional compression over the femoral head consistently achieves excellent hemostasis with minimal complication rates, unlike brachial access. Hence, there is no clinical indication for applying this technique to femoral access.

Before the institution of this technique, a conventional manual compression of the brachial artery access was performed after the procedure. We developed this technical modification, as, like in literature, we had a high rate of brachial artery access related complication, in spite of the technical advantages of being able to do an AVF angioplasty quickly and efficiently. We showed that after incorporation of this technique the incidence of pseudo aneurysms and haematomas reduced significantly. This is a reproducible and easily learnt technique which works on the principle of combining a mechanical barrier just at the level of the arterial adventitia, controlled compression and direct visualization of an occluding plug at the access site.

Manual compression is operator-dependent, lacks real-time visualization, requires prolonged pressure application and limb immobilization, and may delay ambulation and extend hospital stay. While prospective comparative randomized data with alternative closure methods were not collected in this study a comparative historical cohort has been used to reflect our change in practice. Notably, manual compression is less effective for brachial artery access due to the vessel's tendency to slip over the narrow epicondylar surface. In contrast, the described technique enables consistent intimal apposition under ultrasound guidance, with the sheath acting as a temporary extraluminal plug during staged withdrawal.

A recent meta-analysis [8] did suggest that vascular closure devices in retrograde brachial arterial accesses have high technical success rates without increase in adverse events as compared to manual compression. However, there is a lack of published data regarding their use in antegrade brachial artery access.

A limitation of this report is that the exact anti platelet and anticoagulation regimen has not been reported in the baseline characteristics. However, given the large number of patients in each group and given a consistent principle of administering anti platelets and anticoagulation, both before and after adoption of this technique, it is unlikely to be a confounding factor in the results.

## Conclusion

Ultrasound-guided staged sheath withdrawal is a feasible and safe method for closing antegrade brachial artery access, significantly reducing complication rates. This might increase the use of brachial arterial access more routinely, as the risk of subsequent bleeding is considerably reduced. To our knowledge, this technique has not been previously described.

The reproducibility and safety of this ultrasound-guided closure technique may support wider adoption of brachial artery access for neurovascular and complex aortic interventions, intra-aortic balloon pump placement, and intra-arterial hemodynamic monitoring during high-risk anesthesia, domains traditionally constrained by access-site complications. Future prospective studies in these settings and with larger sheath sizes are warranted to validate its role and compare it with conventional closure methods.

## Supplementary Information

Supplementary Material 1. Supplementary Table: Baseline demographic, clinical, and procedural characteristics of both groups: Manual compression group (*n* = 120) and Staged withdrawal group (*n* = 170). Abbreviations: Fr = French; AVF = arteriovenous fistula.

Supplementary Material 2. Supplementary Video: Ultrasound-Guided Staged Sheath Withdrawal for Brachial Artery Access Closure. This video demonstrates the ultrasound-guided staged sheath withdrawal technique for safe and effective closure of antegrade brachial artery access. Key steps include real-time sonographic visualization, gradual sheath retraction under compression, assessment of distal perfusion, and confirmation of hemostasis without hematoma or pseudoaneurysm formation

## Data Availability

Available in the main manuscript. Additional data if required, will be made available on reasonable request.

## Abbreviations

AVF: Arteriovenous fistula
Fr: French
