# Supplementary material for: Ultrasound-guided antegrade brachial artery access closure: a staged withdrawal technique for improved hemostasis
Source: CVIR Endovasc. 2025 Dec 2;8:106. doi: 10.1186/s42155-025-00591-6 (PMC12672984; doi:10.1186/s42155-025-00591-6)
Supplement: Supplementary file 1 — Supplementary Material 1. Supplementary Table: Baseline demographic, clinical, and procedural characteristics of both groups: Manual compression group (n = 120) and Staged withdrawal group (n = 170). Abbreviations: Fr = French; AVF = arteriovenous fistula. [file 42155_2025_591_MOESM1_ESM.docx]

**Supplementary Table:** Baseline demographic, clinical, and procedural characteristics of both groups: Manual compression group (n = 120) and Staged withdrawal group (n = 170).

|  | Manual compression group (n = 120) | Staged withdrawal group (n = 170) |
| --- | --- | --- |
| **Patient characteristics** | | |
| Age (years) | 62.5±10 | 60.45±12.03 |
| Sex, male | 84 | 119 |
| Location of AVF - right: left | 46:74 | 57:113 |
| **Comorbidities** | | |
| Hypertension | 114 | 124 |
| Diabetes mellitus | 70 | 78 |
| Hypothyroid | 10 | 20 |
| Hepatitis C | 4 | 3 |
| Coronary artery disease | 15 | 17 |
| Cerebrovascular accident | 2 | 3 |
| Chronic obstructive pulmonary disease | 2 | 3 |
| Polycystic kidney disease | 5 | 3 |
| **Perianastomotic lesion** | | |
| Stenosis: Occlusion | 65:55 | 88:82 |
| **Brachial artery access specifics** | | |
| Sheath sizes: 5Fr:6Fr | 113:7 | 162:8 |
| Mean diameter of access artery, mm | 4.1 ± 0.5 | 4.2 ± 0.8 |
| Access site calcification | 0 | 0 |

Abbreviations: Fr = French; AVF = arteriovenous fistula;
